# 192. Characteristics of *Staphylococcus aureus* Bacteremia in Patients With Substance Use at a Large Academic Safety-Net Hospital 2020-2021

**DOI:** 10.1093/ofid/ofad500.265

**Published:** 2023-11-27

**Authors:** Christian Gomez, Madhuri Sopirala, Aparna Kambhampati

**Affiliations:** UT Southwestern Medical Center, Dallas, Texas; UT Southwestern Medical Center and Parkland Health, Dallas, Texas; UT Southwestern Medical Center, Dallas, Texas

## Abstract

**Background:**

*Staphylococcus aureus* bacteremia is a serious infection with a high mortality rate. Substance use disorder has been identified as a risk factor and poses unique challenges for treatment and outpatient parenteral antibiotic therapy (OPAT) eligibility. Outcomes of in this patient population have not been well studied. To address this gap, we conducted a retrospective study to evaluate the outcomes of patients with substance use disorder admitted with *S.aureus* bacteremia.

**Methods:**

We conducted a retrospective epidemiological study of adults with substance use within 1 year and admitted to Parkland Hospital from April 2020 to March 2021 with diagnosis of *S. aureus* bacteremia during admission. Clinical and epidemiological data was collected using the electronic medical records system.

**Results:**

68 patients met inclusion criteria. 35 patients (52.4 %) reported active Intravenous drug use (IV). Cocaine use was reported by 40 %, Opioid use by 65.7 %, and amphetamine use by 26.9% of patients. 46% of patients were homeless and 52% were uninsured. Initial sources of bacteremia included skin and soft tissue infections (34%), bone and joint infections (31%), and infective endocarditis (22%). Most patients (40%) were discharged to a facility, while 24% were discharged home and 24% left against medical advice.

Readmission within 30 days was observed in 21% of patients. Mortality at 30 days was 4.5%, while mortality at 1 year was 12%, with 17 patients (25%) lost to follow-up. Infectious complications related to *S. aureus* bacteremia within 1 year were seen in 31% of patients, with SSTI being the most common complication (16.4%). Six patients (9%) were diagnosed with endocarditis in a subsequent encounter. Leaving against medical advice was associated with an increased risk of *S. aureus* related complications after discharge (OR 6.6, 1.69-25 P=0.006)

**Figure:**
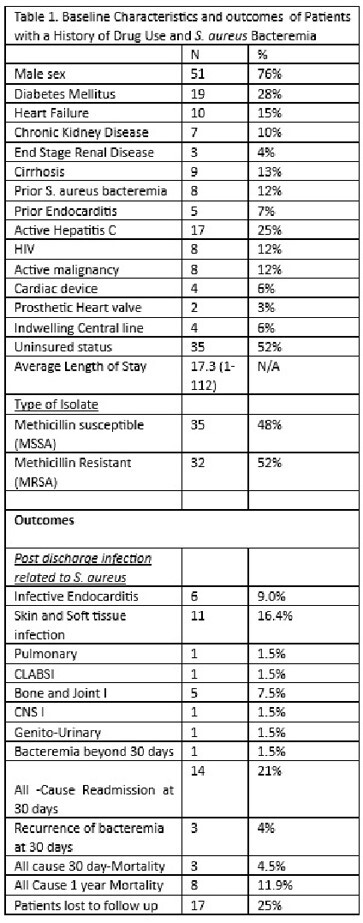

**Figure:**
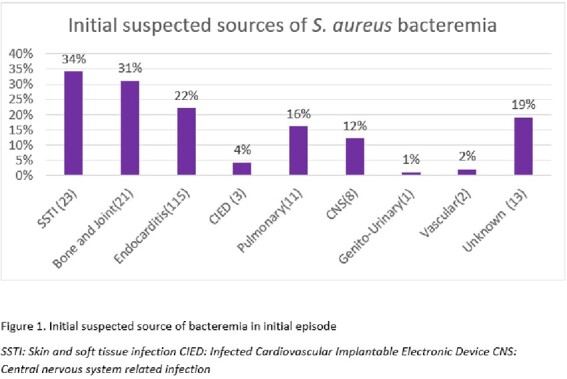

**Figure:**
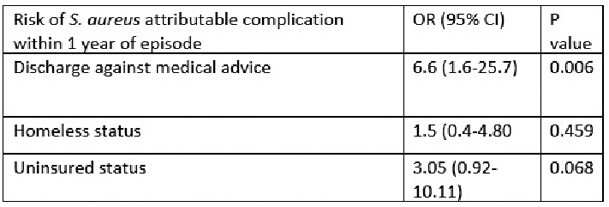

**Conclusion:**

There is significant burden of *S. aureus* bacteremia in patients with substance use disorder. Findings also suggest that infectious complications related to *S. aureus* bacteremia after discharge are common and require close monitoring and timely intervention. There is need for continued research to better understand the outcomes of patients with substance use disorder and to identify strategies for improving care

**Disclosures:**

**All Authors**: No reported disclosures

